# Chemical Composition of Essential Oils from Rare and Endangered Species—*Eryngium maritimum* L. and *E. alpinum* L.

**DOI:** 10.3390/plants9040417

**Published:** 2020-03-30

**Authors:** Małgorzata Kikowska, Danuta Kalemba, Jolanta Dlugaszewska, Barbara Thiem

**Affiliations:** 1 Department of Pharmaceutical Botany and Plant Biotechnology, Poznan University of Medical Sciences, 61-861 Poznań, Poland; bthiem@ump.edu.pl; 2 Institute of Natural Products and Cosmetics, Lodz University of Technology, 90-942 Łódź, Poland; danuta.kalemba@p.lodz.pl; 3 Department of Genetics and Pharmaceutical Microbiology, Poznan University of Medical Sciences, 60-781 Poznań, Poland; jdlugasz@ump.edu.pl

**Keywords:** Sea holly, Alpine eryngo, essential oil composition, germacrene D, organs from intact plant, shoot in vitro cultures, antimicrobial activity of the essential oil

## Abstract

In the present study, the essential oils obtained by hydrodistillation of the organs of *Eryngium maritimum* and *E. alpinum* were analyzed by GC-FID-MS. The dominant constituents assessed in the essential oil of *E. maritimum* were germacrene D (45.2%) in the fruits; hexadecanoic acid (18.5%), menthol (16.8%), and menthone (10.9%) in the roots; 2,3,4-trimethylbenzaldehyde (11.3%) and germacrene D (10.5%) in the leaves; 2,3,4-trimethylbenzaldehyde (11.3%) in the shoot culture. In the case of *E. alpinum*, the main components of the leaf oil were: β-elemenone (10.3%), germacrone (5.8%), two selinadienes (7.1% and 6.7%), and 1,8-cineole (5.3%), which were not found in the oil from shoot culture, whereas the shoot culture oil was dominated by hexadecanoic acid (15.5%), spathulenol (7.5%), (*E*)-β-farnesene (4.9%), germacra-4(15),5,10(14)-trien-1α-ol (4.7%), and falcarinol (4.3%). The essential oils obtained from *E. maritimum* fruits and leaves of the intact plants, measured by the broth microdilution method, were the most active against *T. mentagophytes* and *S. aureus*. Moreover, the essential oil of leaves had the moderate activity against *C. albicans* and *E. coli*. The results showed that the chemical compositions of the essential oils differed decidedly between the two studied species and between the organs. Furthermore, the essential oil of *E. maritimum* may play an important role as antimicrobial agent.

## 1. Introduction

The genus *Eryngium* L., which belongs to the subfamily Saniculoideae of the Apiaceae family, is represented by 230–250 species widespread in Central Asia, America, and Central and Southeast Europe [1]. Among them, there are some species such as *E. campestre*, *E. maritimum*, *E. foetidum*, and *E. creticum* that have been used in the traditional medicines worldwide [2]. The pharmacological activities of *Eryngium* species depend mainly on the presence of triterpenoid saponins, flavonoids, phenolic acids, coumarin derivatives, acetylenes, and the essential oils [3,4].

*E. maritimum* L. (Sea holly) is a rare perennial under strict law protection in Poland and some European countries. It grows on coastal dunes of the Baltic Sea, the Mediterranean basin, and the Black Sea [5]. The phytochemical investigations of *E. maritimum* revealed the presence of secondary metabolites—polyhydroxylated oleanene triterpenoid saponins [6,7], phenolic acids, mostly rosmarinic and chlorogenic acids [8,9], flavonoids containing mainly kaempferol, astragalin, isoquercitrin, rutin, apigenin, and luteolin [8,9,10], coumarins [9], 20-hydroxyecdysone and polypodine B as the main phytoecdysteroids [11], betaines [12], and the essential oil [13,14,15,16,17,18,19]. Several studies concerning the essential oil composition of *E. maritimum* generally investigated aerial parts of the intact plant [13,14,15,16,17,18,19] and no other research dealt with biomass from in vitro cultures. Moreover, the Polish individuals have been examined on the basis of the essential oil characterization for the first time. 

*E. alpinum* L. (Alpine eryngo) is also under strict law protection in the European countries. It is a perennial species that can be found across the European Alps, at the altitudes between 1500 and 2000 m a.s.l. [20]. The phytochemical investigations of *E. alpinum* revealed the presence of secondary metabolites—triterpenoid saponins [21], phenolic acids mainly caffeic acid, rosmarinic acid and its derivative R-(+)-3’-*O*-β-d-glucopyranosyl rosmarinic acid, chlorogenic, iso- and neochlorogenic acids, and 3,4-dihydroxyphenylacetic acid [21,22,23], flavonoids—kaempferol, quercetin, and isoquercetin [21,23,24], and the essential oil [25]. The analysis of the essential oil of the *E. alpinum* aboveground part performed by Dunkić et al. [25], showed the presence of oxygenated sesquiterpenes, sesquiterpene hydrocarbons, oxygenated monoterpenes, and monoterpene hydrocarbons. Falcarinol was also found by Hegenauer [26,27]. To our knowledge, basal leaves as well as biomass from in vitro shoot culture of this species have never been investigated for the presence and the composition of the essential oil. 

The availability of several plant species is limited due to their protection status. Increasing pollution and adverse changes occurring in the natural environment result in the shrinkage of plant resources, and the collection of the raw materials from fields in such regions becomes questionable. The alternative solution to these restrictions may be production of plant biomass of the endangered species applying the biotechnological methods. Rapid production of homogeneous and renewable biomass capable of biosynthesis of substances with the pharmacological activity, and most importantly the possibility of strict control of this process, indicate the opportunity for wider application of the biotechnological methods in the pharmaceutical and cosmetic industry [28,29].

The aim of this work was to study the chemical composition of the essential oils obtained from different parts of the intact plants as well as in vitro shoot cultures of *E. maritimum* L. and *E. alpinum* L., and to screen the antimicrobial activity of the essential oils obtained from mature fruits and basal leaves of the *E. maritimum* intact plant against Gram-positive bacterium *Staphylococcus aureus*, Gram-negative bacterium *Escherichia coli*, yeast *Candida albicans*, and dermatophyte fungus *Trichophyton mentagrophytes*. Our study on the essential oil composition of *E. maritimum* has completed the existing knowledge on this subject and supplemented the raw materials not yet tested, and thus not characterized, especially those obtained by the biotechnological methods under controlled conditions. In addition, our study has provided the first preliminary results of the analysis of the *E. alpinum* essential oil from the raw materials obtained from the intact plant and biomass from in vitro culture. Thus, in vitro cultures of these endangered and protected species can be the alternative source of the raw material for phytochemical examination and the biological activity studies. In addition, this has been the first report on the content and the composition of the essential oils from *Eryngium* species growing in Poland—*E. maritimum* on Polish beaches and *E. alpinum* in a botanical garden.

## 2. Results and Discussion

**Figure 1:**
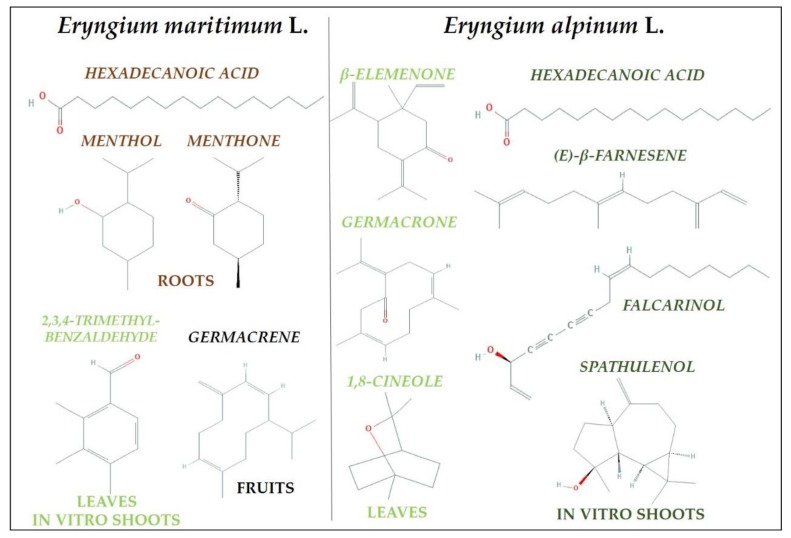
The main compounds of the essential oil from different organs of the intact plants and in vitro shoot cultures of *E. maritimum* and *E. alpinum*.

The main compounds of *E. maritimum* and *E. alpinum* essential oils are illustrated in Figure 1.

Hydrodistillation of the dried parts (fruits, leaves, and roots) and in vitro-derived shoot culture of *E. maritimum* gave the essential oils in the yield of 0.30%, 0.06%, 0.01%, and 0.01%, respectively. These values have been in accordance with the earlier data that reported 0.31–0.93% for the fruit [18] and 0.06–0.13% for the aerial part [17] of the essential oil of this species.

The composition of the essential oils was analyzed by GC-FID-MS. The identified compounds of *E. maritimum* oils are presented in Table 1.

In total, more than fifty constituents were identified in each oil. The composition of the intact plant essential oils differed significantly both between plant parts and from the earlier data. However, some similarities were observed in both regards. The main constituents of the leaf and fruit oils were sesquiterpenes and this is in good agreement with the previous reports [14,15,16,17,18]. The content of the dominant compound of this group, germacrene D, amounted to 45.2% in the fruit oil and 10.5% in the leaf oil. Other important constituents were γ-elemene (6.9%) and β-ylangene (4.0%) in the fruit oil, as well as 2,3,4-trimethylbenzaldehyde (11.3%), spathulenol (4.9%), and neophytadiene (5.2%) in the leaf oil. In the leaf oil, the mixture of two sesquiterpene aldehydes 4βH-cadin-9-en-15-al and 4βH-muurol-9-en-15-al (11.5%) was tentatively identified on the basis of the retention index (RI) because mass spectra were not available. These compounds, together with the corresponding alcohols, were previously isolated from the *E. maritimum* oil and identified by NMR [16]. Darriet et al., [17] analyzed the essential oil of leaves, flowers, stems, and roots and compared the composition of the several oil samples of *E. maritimum* from Corsica and Sardinia. They observed small differences in the composition of the essential oils isolated from aerial parts of the plant and stated that the dominant components were germacrene D (32.1–42.5%), 4βH-cadin-9-en-15-al (18.4–27.6%), 4βH-cadin-9-en-15-ol (2.2–10.5%), and 4βH-muurol-9-en-15-al (4.3–9.3%). The main difference between 12 oil samples was the higher content of germacrene D in the Corsican samples (32.2–45.9%) than in the Sardinian ones (13.7–23.8%) [17]. Moreover, the high content of germacrene D (40%) was found in the essential oil of the Portuguese sample [13,14]. In the aerial part oil from the Italian species, the content of germacrene D was similar (10.4%) to the presented study (10.5%) [14]. The high amount of germacrene D (13.6–31.7%) was also noticed previously in the fruit essential oil of *E. maritimum* growing on the Tunisian shoreline. The major individual compounds, except germacrene D, were 15-hydroxy-α-muurolene (12.0–18.6%) and germacrene B (6.8–15.0%) [18].

The root essential oil differed significantly from the fruit and leaf oils. It contained mainly oxygenated monoterpenes menthol (16.8%), menthone (10.9%), and menthyl acetate (5.6%), as well as 2,3,4-trimethylbenzaldehyde (5.6%). According to the research of Darriet et al., [17], the root oil of this species also differed entirely from the essential oil of the aerial part. However, its composition was different from the presented oil. It contained 2,4,5- (39.8%) and 2,3,6-trimethylbenzaldehyde (29.0%), α-muurolene (23.5%), and germacrene D (2%). Various trimethylbenzaldehyde isomers (2,3,4-trimethylbenzaldehyde, 2,4,5-trimethylbenzaldehyde, 2,3,6-trimethylbenzaldehyde, and 2,4,6-trimethylbenzaldehyde) were reported in high concentrations in the essential oil from different parts of *E. maritimum* [16,17,18,19] and many other *Eryngium* species [30]. Unambiguous identification of these isomers is difficult because of the similarity of their mass spectra and different literature data of RIs. In this paper, identification was done on the basis of MassFinder 4.1.

The composition of the in vitro shoot essential oil was very similar to the leaf oil. The majority of the identified compounds were found in both oils. What is more, the content of the constituents was similar, with the exception of germacrene D, the content of which was lower in the shoot oil than in the leaf oil (3.8% versus 10.5%). The most important feature that differentiated these oils was the presence of the pronounced amounts (1.2–5.7%) of some sesquiterpenes in the shoot oil, which were found neither in the leaf oil nor in the other two oils, namely hydrocarbons with eremophilane and selinane skeleton, (*E*)-nerolidol, and two ketones β-elemenone and germacrone. The absence of hexanal, octanal, and undecan-2-one was noticed in the shoot oil in contrast to the in vitro shoot oil.

The essential oils from basal leaves of the intact plant and in vitro shoot culture of *E. alpinum* were obtained with the yield of 0.01%. The constituents of these oils are presented in Table 2.

The dominant constituents of the *E. alpinum* essential oils were sesquiterpenes, both hydrocarbons and oxygenated derivatives. Although leaf and in vitro shoot oils have numerous common components, they differed significantly. The main components of the leaf oil were β-elemenone (10.3%), germacrone (5.8%), two selinadienes (7.1% and 6.7%), and 1,8-cineole (5.3%) that were not found in the in vitro shoot oil. On the other hand, the in vitro shoot oil was dominated by hexadecanoic acid (15.5%), spathulenol (7.5%), germacra-4(15), 5,10(14)-trien-1α-ol (4.7%), (*E*)-β-farnesene (4.9%), and falcarinol (4.3%). Each of these components was present in the leaf oil, however, in a smaller amount. In respect to the presence of the four mentioned sesquiterpenes (ketones and selinadienes), (*E*)-nerolidol, and the low content of germacrene D (1.7%), the leaf oil of *E. alpinum* resembled the in vitro shoot oil of *E. maritimum* (Table 1). The composition of the leaf essential oil is quite different than that previously reported for the aerial part oil, which contained caryophyllene oxide (21.6%), bicyclogermacrene (11.8%), germacrene D (10.3%), and α-bisabolol (7.8%) [25] as the major constituents.

The study on the essential oil composition is a part of a bigger project aiming at phytochemical screening of *Eryngium* species: protected *E. maritimum* and *E. alpinum* as well as rare *E. planum* and *E. campestre*. In comparison with our previous investigation on the essential oils from different parts (inflorescence, stalk leaves, rosette leaves, and roots) as well as shoot in vitro culture of *E. planum*, some differences were observed between the aerial part, root, and in vitro shoot oils [30]. This is the first time the essential oils of the intact plant and shoot in vitro culture of *E. maritimum* and *E. alpinum* have been comparatively analyzed. It should be pointed out that the composition of the essential oil produced by in vitro shoot culture of *E. maritimum* (Table 1) was similar to the composition of the basal leaf oil. On the contrary, *E. alpinum* (Table 2) and *E. planum* [30] shoot cultures produced different oils than any aerial part. The essential oil composition of *E. campestre* aerial parts at the flowering stage [31,32,33,34] and roots [34] was the object of many studies.

The activity of the essential oils obtained from *E. maritimum* basal leaves and fruits was evaluated by the broth microdilution method. Both oils were the most active against *T. mentagrophytes* (Minimal Inhibitory Concentration MIC = 1.56 ± 0.0 mg/mL and 7.5 ± 0.0 mg/mL, respectively) and *S. aureus* (MIC = 12.5 ± 0.0 mg/mL and 60 ± 0.0 mg/mL, respectively). The essential oil from basal leaves had also the moderate activity against *C. albicans* (MIC = 12.5 ± 0.0 mg/mL) and *E. coli* (MIC = 25 ± 0.0 mg/mL) (Table 3).

The studies on the antimicrobial activity of the essential oil and its oxygenated sesquiterpene fractions from the aerial part of *E. maritimum* estimated by the agar diffusion method against several bacteria revealed the significant effect of 4βH-cadin-9-en-15-al, germacrene D, 4βH-cadin-9-en-15-ol (8.3%), and 4βH-muurol-9-en-15-al mixture against *Escherichia coli*, *Enterococcus faecalis*, and *Listeria monocytogenes* [15]. Moreover, the essential oil from the aerial part of this species growing in Portugal showed the moderate activity against *Leishmania infantum* promastigotes growth and no activity against *Giardia lamblia* [13,14].

The interest in the essential oils obtained from the *Eryngium* taxa has been increasing since some of the constituents and the essential oils showed the antimicrobial activities, for example *E. creticum*, *E. campestre*, *E. thorifolium*, and *E. duriaei* [4]. In the disc diffusion method, the essential oil of *E. thorifolium*, rich in α-pinene, showed the antibacterial activity against *S. aureus* with the inhibition zone ranging from 13 to 19 mm, which referred to sensitivity of the test microorganisms [32]. Moreover, the essential oil of *E. duriaei* revealed the antifungal activity against *T. mentagrophytes*, which was probably related to the caryophyllene derived compounds [35].

The antimicrobial activity of *E. maritimum* as well as *E. planum* and *E. campestre* has been already studied [36,37]. However, it was the extracts and their fractions that were examined, not the essential oils. The crude ethanolic extracts of both leaves and roots of investigated *E. maritimum* showed the significant antifungal activity against *T. mentagrophytes* and the moderate antibacterial activity against *S. aureus* [36]. This observation was in a great agreement with the study of Kholkhal [8], who showed the moderate antibacterial activity of the methanolic extract from the *E. maritimum* root against *S. aureus*. The methanolic extract of roots and saponin-phenolic acid fraction of this extract were the most active against *C. albicans* [37]. The results of Meot-Duros studies on the *E. maritimum* antimicrobial activity of the leaf hydromethanolic extracts, measured by microdilution method, showed that apolar fractions were more active than polar fractions. The antibacterial activity of apolar fraction against *S. aureus* showed MIC value of 10 µg/mL and the antifungal activity of apolar fraction against *C. albicans* showed MIC value of 100 µg/mL, while polar fractions had no antimicrobial activity. Moreover, both apolar and polar fractions were not sensitive to *E. coli* [38].

## 3. Materials and Methods 

### 3.1. Intact Plants

The primary explants of *E. maritimum* and *E. alpinum* were collected from Adam Mickiewicz Botanical Garden in Poznań, Poland in September 2017. The voucher specimens have been deposited in the Department of Pharmaceutical Botany and Plant Biotechnology of Poznan University of Medical Sciences under the numbers: H-M-2017-101 (*E. maritimum*) and H-AP-2017-102 (*E. alpinum*). The permission for the collection of the organs from the protected species in order to use them for scientific research was granted by the Regional Director for Environmental Protection in Poznan. 

### 3.2. In Vitro Shoot Culture

For the initiation of aseptic culture of *E. maritimum* and *E. alpinum*, the shoot fragments with axillary buds isolated from plantlets were used as explants. The isolated primary explants were rinsed in distilled water for 5 min and dipped in 70% (*v*/*v*) ethanol-water solution for 30 s followed by rinsing in 1.33% (*E. maritimum*) or 2.5% (*E. alpinum*) sodium hypochloride solution, containing two drops of surfactant Tween 80 for 8 min. They were finally rinsed three times in sterilized double-distilled water.

The explants of both the species were placed in a Erlenmeyer flask with 50 mL of the solidified MS medium (Murashige and Skoog [39]) with plant growth regulators—benzylaminopurine (BAP; Sigma-Aldrich, Saint Louis, MO, USA), indole-3-acetic acid (IAA; Sigma-Aldrich, Saint Louis, MO, USA), and gibberellic acid (GA_3_; Sigma-Aldrich, Saint Louis, MO, USA), each at the concentration of 1.0 mg/L [23,40]. The culture vessels were placed in a growth chamber (21 ± 2 °C; with a 16 h light/8 h dark photoperiod; 55 µmol/m^2^∙s light) and subcultured every 5 weeks. Multiplication of shoots via the axillary branching method on MS medium was repeated many times, using at least 10 explants per repetition. Shoots were air dried. 

### 3.3. Isolation and the Analysis of the Essential Oil

In the present study, mature fruits, basal leaves, and roots of the intact plant, as well as biomass from in vitro shoot culture of *E. maritimum*, were chosen for the analysis of the essential oil. Furthermore, the preliminary investigation of the chemical composition of the essential oil from basal leaves and in vitro shoot culture was conducted for *E. alpinum*.

The essential oils were obtained by three-hour hydrodistillation of the dried plant material using the glass Clevenger-type apparatus, according to European Pharmacopoeia 5.0. [30].

The GC-FID-MS analyses were performed using the Trace GC Ultra apparatus (Thermo Electron Corporation, Waltham, MA, USA) with FID and MS DSQ II detectors and the FID-MS splitter (SGE). Operating conditions: apolar capillary column Rtx-1ms (Restek), 60 m × 0.25 mm i.d., film thickness 0.25 µm; temperature program, 50–300 °C at 4 °C/min; SSL injector temperature 280 °C; FID temperature 300 °C; split ratio 1:20; carrier gas helium at a regular pressure 200 kPa. Mass spectra were acquired over the mass range 30–400 Da, ionization voltage 70 eV; ion source temperature 200 °C.

Identification of components was based on the comparison of their MS spectra with those of laboratory-made MS library, commercial libraries (NIST 98.1, Wiley Registry of Mass Spectral Data, 8th Ed. and MassFinder 4.1, Hamburg, Germany) and with the literature data [41,42] along with the retention indices (Rtx-1, MassFinder 4.1) associated with a series of alkanes with linear interpolation (C8-C26). The quantitative analysis (expressed as percentages of each component) was carried out by peak area normalization measurements without correction factors.

### 3.4. The Antimicrobial Assay

The essential oils from basal leaves and fruits of the intact plant of *E. maritimum* were tested against selected bacteria and fungi strains.

### 3.5. Preparing of the Essential Oils

The essential oils were dissolved in DMSO and then diluted in MHB (bacteria) or SDB (fungi) to a concentration of 50 mg/mL—the leaf oil, and 240 mg/mL—the fruit oil. The final concentration of DMSO not excided 0.1%. The solutions of the essential oils (100 µL) were two-fold serially diluted with MHB (bacteria) and SDB (fungi) in 96 well microtiter plates to the concentrations ranging from 50 mg/mL to 0.10 mg/mL—the leaf oil, and from 240 mg/mL to 0.47 mg/mL—the fruit oil. Simultaneously, a test was carried out determining the antimicrobial activity of DMSO used for the preparation of the stock solution of the essential oils.

### 3.6. Microorganisms

The following microorganisms were tested: Gram-positive strain *S. aureus* ATCC 25923; Gram-negative strain *E. coli* ATCC 25922; yeast *C. albicans* ATCC 10231 and dermatophyte *T. mentagrophytes* ATCC 9533. All the strains were purchased from the American Type Culture Collection. Amikacin (Sigma-Aldrich, USA; A0368000) and nystatin (Sigma-Aldrich, USA; N4014) were used as reference substances for bacteria and fungi respectively (amikacin—breakpoint for *Enterobacterales* and *S. aureus* according to EUCAST: S ≤ 8, R > 8; nystatin—there is no breakpoint limit range for microdilution tests).

The bacteria were grown in the Brain Heart Infusion broth (BHI, Oxoid, UK) and *C. albicans* in Sabouraud dextrose broth (SDB; Oxoid, UK, company, city, state if USA, country) at 34 °C for 18 h. After incubation, 2 mL of each culture was harvested by centrifugation (3000 rpm for 15 min), re-suspended in 1.5 mL of 10 mM phosphate buffered saline (PBS, pH 7.0, Sigma-Aldrich, USA), and then diluted in a suitable medium (bacteria in Mueller-Hinton broth, MHB OXOID, UK; fungi in SDB).

Filamentous fungi were inoculated on the Sabouraud dextrose agar (SDA; OXOID, UK) and incubated at 34 °C for adequate formation of conidia. The culture of the *T. mentagrophytes* were covered with a sterile 0.9% NaCl solution supplemented with 0.1% Tween 80. Then the conidia were carefully rubbed with a cotton swab and transferred to a sterile flask. The suspension was homogenized, vortexed, and filtered. The number of conidia (macroconidia and microconidia) in the suspension was determined using haemocytometer chamber as well as the serial dilution agar plating method. Inoculum was examined for the presence of hyphae, and both macroconidia and microconidia were observed.

Bacterial and fungal suspensions were diluted in suitable broth (bacteria in Mueller–Hinton broth; MHB OXOID, UK; fungi in SDB) to obtain the final suspension containing about 1 × 10^6^ CFU/mL (bacteria), 1 × 10^5^ CFU/mL (*C. albicans*), and 5 × 10^5^ CFU/mL (*T. mentagrophytes*).

### 3.7. Evaluation of Minimal Inhibitory Concentration and Minimal Bactericidal/Fungicidal Concentratioin

The minimal inhibitory concentration (MIC) of the essential oils was determined by employing the broth microdilution method in 96-well plates following EUCAST with some modifications for essential oils [43]. One hundred microliters of each dilution of the essential oil and sterile nutrient broth (for growth control) were distributed into the wells of the micro titer plates. Each well was inoculated with 100 µL of a microbial suspension. The final concentration of microorganisms was determined for all the strains by subculture of the growth control on TSA (bacteria) and SDA (fungi) plates.

All the microtiter plates were incubated at 34 ± 1 °C for 18 h (bacteria and *C. albicans*) and for 72 h (*T. mentagrophytes*). The MICs values were recorded as the lowest concentration of the essential oils that inhibited visible growth of the tested microorganisms. In order to determine the minimal bactericidal/fungicidal concentration (MBC/MFC), after recording the MIC endpoint, the concentrations equal to and higher than MIC were subcultured (10 µL) on Trypticase Soy Agar (TSA; OXOID, UK)—bacteria, and SDA—fungi. The MBC was defined as the lowest concentration at which no growth was observed. All the tests were performed in duplicate and the antimicrobial activity was expressed as mean values ± standard deviation (SD).

## 4. Conclusions

Our study showed that different parts of *Eryngium* species from intact plants and in vitro shoot cultures showed different yields and compositions of the essential oils. Comparing the volatile constituents of *E. maritimum*, *E. alpinum*, and other *Eryngium* species, it can be stated that there is a group of compounds that are common for the majority of the essential oils. Among them there are mono- and sesquiterpene hydrocarbons, and especially germacrene D, widespread as the components of the essential oils as well as compounds rarely found in the essential oils such as *cis*-chrysanthenyl acetate, trimethylbenzaledydes, and falcarinol. Our study on the essential oil composition of *E. maritimum* and *E. alpinum* has completed the existing knowledge on this subject. It was shown that in vitro shoot culture of *E. maritimum* produced the essential oil with the composition similar to the composition of the intact plant and *E. alpinum* basal leaves and shoot culture gave significantly different essential oils. Thus, in vitro cultures of these endangered and protected species can be the alternative source of the raw material for phytochemical examination and the biological activity studies. In addition, this has been the first report on the content and the composition of the essential oils from *Eryngium* species growing in Poland. The essential oils obtained from *E. maritimum* fruits and basal leaves of the intact plant were the most active against dermatophyte fungus *T. mentagophytes* and Gram-positive bacterium *S. aureus*. Moreover, the essential oil of basal leaves had also the moderate activity against *C. albicans* and Gram-negative *E. coli*. 

To sum up, it is necessary to take into account in the above comparative analyses not only the ecotype and geographical localization of the plants but also the type of part of the plant analyzed, as well as the fact whether the oils come from the ground plant or in vitro cultures.

## Figures and Tables

**Table 1:** The chemical composition (%) of the essential oil from different organs of the intact plant and in vitro shoot culture of *E. maritimum*.

Constituent	RI_exp_	RI_lit_	R	F	L	S
Hexanal	775	777	-	-	0.1	-
2-Butylfuran	870	869	-	t	0.1	t
Heptanal	879	882	0.3	-	t	-
α-Pinene	932	936	0.2	t	0.2	0.5
Thuja-2,4(10)-diene	946	946	0.1	-	0.1	0.6
β-Pinene	973	978	0.2	-	t	t
Mesitylene	977	980	0.6	0.1	0.9	3.5
Octanal	978	0981	1.1	-	0.1	-
α-Phellandrene	1000	1002	0.1	-	-	-
1,2,3-Trimethylbenzene	1003	1005	0.2	-	0.4	1.0
*p*-Cymene	1013	1015	t	t	t	0.2
1,8-Cineole	1021	1024	0.3	t	t	t
Limonene	1025	1025	0.4	-	t	0.3
1-Methoxy-2-(1-methylethenyl)benzene	1076	1074	-	-	0.1	0.3
6-Camphenone	1079	1082	-	-	0.2	0.5
α-Campholenal	1103	1105	t	t	0.1	t
*cis*-Verbenol	1128	1132	-	t	0.2	0.9
Menthone	1133	1136	10.9	-	-	-
*trans*-Verbenol	1133	1137	-	0.1	0.6	3.6
*p*-Mentha-1,5-dien-8-ol	1144	1142	0.2	t	0.3	0.9
Isomenthone	1145	1146	1.8	-	-	-
Neomenthol	1152	1156	0.8	-	-	-
Terpinen-4-ol	1161	1164	-	-	-	0.2
Menthol	1170	1172	16.8	-	-	-
Myrtenal	1169	1172	0.3	-	t	0.3
α-Terpineol	1176	1178	-	-	-	0.1
Verbenone	1180	1183	-	-	-	0.4
Pulegone	1214	1215	1.1	-	-	-
Piperytone	1228	1226	0.3	-	-	-
*cis*-Chrysanthenyl acetate	1248	1253	0.2	2.3	0.7	0.7
Undecan-2-one	1275	1273	-	-	0.1	-
Menthyl acetate	1278	1280	5.3	-	-	-
Dihydroedulan II	1280	1284	0.2	t	0.5	0.4
2,3,6-Trimethylbenzaldehyde	1288	1287	1.5	0.2	2.0	1.8
2,3,4-Trimethylbenzaldehyde	1330	1331	5.6	1.1	11.3	11.3
Bicycloelemene	1338	1338	-	0.9	-	-
α-Cubebene	1353	1355	-	0.2	0.1	0.1
(*E*)-β-Damascenone	1363	1363	-	-	0.1	0.2
α-Ylangene	1376	1376	-	0.1	-	-
α-Copaene	1380	1379	t	0.8	0.9	0.4
β-Bourbonene	1385	1387	0.3	0.2	1.1	0.3
β-Elemene	1387	1389	1.3	1.7	0.9	0.5
β-Ylangene	1418	1420	-	4.0	1.2	t
β-Caryophyllene	1421	1421	2.9	0.2	0.4	0.8
γ-Elemene	1432	1429	0.2	6.9	t	0.9
β-Copaene	1429	1431	0.2	2.4	1.2	t
γ-Decanolide	1432	1433	0.3	-	-	-
Isogermacrene D	1443	1445	-	0.8	0.2	t
(*E*)-β-Famesene	1447	1447	0.5	t	t	0.6
Selina-4(15),6-diene	1450	1450	-	0.9	t	0.2
α-Humulene	1451	1453	0.2	0.7	0.7	t
β-Ionone	1468	1468	-	-	0.3	t
*ar*-Curcumene	1470	1473	0.4	-	-	-
γ-Muurolene	1473	1475	t	t	t	0.3
Germacrene D	1476	1480	2.6	45.2	10.5	3.8
Aristolochene	1484	1486	0.2	-	0.4	2.2
*cis*-β-Guaiene	1488	1488	0.6	-	-	-
Eremophila-1(10),7-diene	1489	1488	-	-	-	5.7
Valencene	1493	1494	-	-	1.9	-
Bicyclogermacrene	1494	1494	0.3	1.5	-	-
α-Muurolene	1495	1496	-	-	0.3	t
ε-Amorphene	1498	1498	-	0.8	-	-
(*E,E*)-α-Farnesene	1501	1498	0.2	-	t	0.2
Eremophila-1(10),8,11-triene	1507	1504	-	-	-	1.2
γ-Cadinene	1509	1507	0.2	1.3	0.4	t
Calamenene	1510	1517	0.1	0.1	t	0.2
δ-Cadinene	1515	1520	0.4	2.3	1.0	2.0
α-Calacorene	1531	1527	-	0.3	0.3	t
Selina-4(15),7(11)-diene	1531	1534	-	-	-	2.0
Elemol	1537	1541	-	1.0	-	-
Selina-3,7(11)-diene	1539	1542	-	-	-	1.6
Salviadienol	1543	1545	-	0.6	0.7	0.5
Germacrene B	1552	1552	-	1.8	-	-
(*E*)-Nerolidol	1552	1553	-	-	-	1.9
Mintoxide	1561	1565	-	0.4	0.5	1.0
Spathulenol	1564	1572	0.6	-	4.9	1.6
Germacrene D-4-ol	1570	1576	-	4.2	-	-
Caryophyllene oxide	1570	1578	0.6	-	0.3	t
β-Elemenone	1586	1589	-	-	-	2.4
Salvial-4(14)-en-1-one	1586	1592	0.1	1.0	2.4	1.3
Globulol	1589	1592	0.6	-	-	-
Torilenol	1597	1599	-	0.9	2.1	2.1
T-muurolol	1629	1633	0.6	1.2	-	-
α-Cadinol	1642	1642	1.0	1.8	-	-
Cadina-4,10(14)-dien-1α-ol	1664	1662	-	-	-	0.5
Eudesma-4(15),7-dien-1β-ol	1673	1671	-	2.8	-	-
4βH-Cadin-9-en-15-al 4βH-Muurol-9-en-15-al	1686	1684	-	2.3	11.5	3.0
	1686	1684				
Ferulyl angelate	1680	1682	0.2	-	-	0.6
Germacrone	1680	1684	-	-	-	1.5
Tetradecanoic acid	1749	1748	0.4	-	-	-
Nootkatone	1781	1782	-	-	1.5	4.0
Neophytadiene 2	1836	1830	0.5	0.7	5.2	1.7
Hexadecanoic acid	1967	1968	18.5	0.8	0.5	4.0
Falcarinol	2000	2000	1.3	-	-	-
γ-Hexadecanolide	2070	2081	0.4	-	-	0.4
Phytol	2101	2114	-	-	2.2	2.3
Total identified	-	-	84.2	94.6	71.7	79.5
Monoterpene hydrocarbons (MH)	-	-	1.2	0	0.7	2.6
Oxygenated monoterpenes (MO)	-	-	38.0	2.4	2.1	7.6
Sesquiterpene hydrocarbons (SH)	-	-	10.6	73.1	21.5	23.0
Oxygenated sesquiterpenes (SO)	-	-	3.5	16.2	23.9	19.8
Others (O)	-	-	30.9	2.9	23.5	26.5
The essential oil yield	-	-	0.01	0.30	0.06	0.01

RI_exp_—Experimental Retention Index, RI_lit_—Literature Retention Index, R—Roots of Intact Plant, F—Fruits of Intact Plant, L—Basal Leaves of Intact Plant, S—In Vitro Shoot Culture, t—Trace (percentage value less than 0.05%).

**Table 2:** The chemical composition (%) of the essential oil from basal leaves of the intact plant and in vitro shoot culture of *E. alpinum*.

Constituent	RI_exp_	RI_lit_	L	S
Hexanal	777	777	-	0.2
Heptanal	878	881	-	0.3
α-Pinene	932	936	1.4	0.1
Camphene	945	950	0.2	-
β-Pinene	975	978	0.2	0.1
2-Pentylfuran	977	981	0.2	0.3
Octanal	978	981	-	3.0
Myrcene	984	987	0.4	-
α-Phellandrene	999	1002	0.2	-
*p*-Cymene	1012	1015	0.5	-
1,8-Cineole	1019	1024	5.3	-
Limonene	1023	1025	1.1	0.2
γ-Terpinene	1048	1051	0.2	-
Octanol	1060	1063	-	0.1
Nonan-2-one	1072	1074	-	0.2
Nonanal	1079	1076	-	0.5
Fenchol	1097	1099	0.1	-
*cis*-Verbenol	1130	1132	0.1	0.2
Menthone	1133	1136	0.6	-
Borneol	1147	1150	0.5	-
Neomenthol	1157	1156	0.6	-
Octanoic acid	1168	1166	0.1	0.3
Terpinen-4-ol	1161	1164	0.9	-
α-Terpineol	1172	1176	1.1	-
Citronellol	1213	1213	0.5	-
*cis*-Chrysanthenyl acetate	1248	1253	0.6	2.1
Bornyl acetate	1267	1270	0.4	0.2
Menthyl acetate	1277	1280	0.2	-
Dihydroedulan II	1282	1280	0.2	0.7
2,3,4-Trimethylbenzaldehyde	1327	1331	2.5	1.0
Terpinyl acetate	1331	1335	1.7	0.2
Citronellyl acetate	1334	1337	0.2	-
β-Damascenone	1361	1363	t	0.1
α-Copaene	1376	1379	0.7	0.6
β-Bourbonene	1385	1387	0.1	0.3
β-Elemene	1386	1389	0.5	1.7
*cis*-α-Bergamotene	1409	1411	-	0.5
β-Caryophyllene	1419	1421	1.4	1.7
γ-Elemene	1427	1429	1.4	1.6
*trans*-α-Bergamotene	1432	1434	t	0.6
Erythrodiene	1444	1446	0.3	2.1
(*E*)-β-Farnesene	1447	1446	0.7	4.9
*trans*-β-Ionone	1464	1468	0.5	0.4
*ar*-Curcumene	1470	1473	-	2.2
γ-Muurolene	1472	1475	0.3	0.2
Germacrene D	1475	1479	1.7	2.5
(*Z,E*)-α-Farnesene	1481	1480	0.8	1.2
α-Muurolene	1494	1496	0.5	1.9
(*E,E*)-α-Farnesene	1498	1498	-	1.8
γ-Cadinene	1505	1507	0.3	0.9
Calamenene	1509	1517	0.7	-
δ-Cadinene	1514	1520	3.8	1.8
Selina-4(15),7(11)-diene	1530	1534	7.1	-
Selina-3,7(11)-diene	1538	1542	6.7	-
Salviadienol	1541	1545	t	1.5
1.5(*E*)-Nerolidol	1550	1553	2.5	-
Spathulenol	1568	1572	1.7	7.5
Caryophyllene oxide	1574	1578	1.9	2.2
Elemenone	1583	1589	10.3	-
Salvial-4(14)-en-1-one	1588	1592	1.1	1.3
Torilenol	1596	1597	0.8	2.0
1,10-di-*epi*-Cubenol	1615	1615	1.5	-
β-Bisabolol	1656	1659	-	1.5
Germacra-4(15), 5,10(14)-trien-1α-ol	1669	1680	1.7	4.7
Germacrone	1675	1684	5.8	-
Tetradecanoic acid	1749	1748	1.3	-
Hexahydrofarnesyl acetone	1829	1817	2.6	3.9
Neophytadiene 2	1835	1836	1.8	1.4
Hexadecanoic acid	1951	1968	4.3	15.5
Falcarinol	1998	2000	3.1	4.3
Phytol	2099	2114	2.0	1.9
Pentacosane	2499	2500	0.3	1.0
Hexacosane	2599	2600	0.1	0.3
Heptacosane	2696	2700	0.4	0.3
Nonacosane	2890	2900	0.9	0.3
Total Identified			91.6	86.3
Monoterpene hydrocarbons (MH)	-	-	4.2	0.4
Oxygenated monoterpenes (MO)	-	-	13.0	3.5
Sesquiterpene hydrocarbons (SH)	-	-	27.0	26.5
Oxygenated sesquiterpenes (SO)	-	-	27.3	20.7
Others (O)	-	-	20.1	35.2
The essential oil yield	-	-	0.01	0.01

RI_exp_—Experimental Retention Index, RI_lit_—Literature Retention Index, L—Basal Leaves of Intact Plant, S—In Vitro Shoot Culture, t—Trace (percentage value less than 0.05%).

**Table 3:** The activity of the essential oils from *E. maritimum* leaves and fruits against standard microbial strains.

Microorganism	Leaf Oil		Fruit Oil		Amikacin		Nystatin	
	MIC ± SD [mg/mL]	MBC ± SD [mg/mL]	MIC ± SD [mg/mL]	MBC ± SD [mg/mL]	MIC ± SD [µg/mL]	MBC ± SD [µg/mL]	MIC ± SD [µg/mL]	MFC ± SD [µg/mL]
*S. aureus* ATCC 25923	12.5 ± 0.0	>25	60 ± 0.0	>120	2.0 ± 0.0	2.0 ± 0.0	-	-
*E. coli* ATCC 25922	25 ± 0.0	>25	>120	>120	2.0 ± 0.0	2.0 ± 0.0	-	-
*C. albicans* ATCC 10231	12.5 ± 0.0	25 ± 0.0	>120	>120	-	-	8.0 ± 0.0	16.0 ± 0.0
*T. mentagrophytes* ATCC 9533	1.56 ± 0.0	3.13 ± 0.0	7.5 ± 0.0	11.25 ± 5.3	-	-	8.0 ± 0.0	64.0 ± 0.0

MIC—Minimal Inhibitory Concentration; MBC—Minimal Bactericidal Concentration; MFC—Minimum Fungicidal Concentration; SD—Standard Deviation.

## References

[1] Wörz A. (2004). On the distribution and relationships of the south-west Asian species of *Eryngium* L. (Apiaceae-Saniculoideae). Turk. J. Bot..

[2] Kupeli E., Kartal M., Aslan S., Yesilada E. (2006). Comparative evaluation of the anti-inflammatory and antinociceptive activity of Turkish *Eryngium* species. J. Ethnopharmacol..

[3] Wang P., Su Z., Yuan W., Deng G., Li S. (2012). Phytochemical constituents and pharmacological activities of *Eryngium* L. (Apiaceae). Pharm. Crops.

[4] Erdem S.A., Nabavi S.F., Orhan I.E., Daglia M., Izadi M., Nabavi S.M. (2015). Blessings in disguise: A review of phytochemical composition and antimicrobial activity of plants belonging to the genus *Eryngium*. Daru J. Pharm. Sci..

[5] Kikowska M., Thiem B., Ramawat K., Ekiert H., Goyal S. (2020). In Vitro Systems of Selected Eryngium Species (*E. planum*, *E. campestre*, *E. maritimum*, and *E. alpinum*) for Studying Production of Desired Secondary Metabolites (Phenolic Acids, Flavonoids, Triterpenoid Saponins, and Essential Oil). Plant Cell and Tissue Differentiation and Secondary Metabolites. Reference Series in Phytochemistry.

[6] Hiller K., von Mach B., Franke P. (1976). Saponins of *Eryngium maritimum* L. Part 25. Contents of various Saniculoideae. Pharmazie.

[7] Kowalczyk M., Masullo M., Thiem B., Piacente S., Stochmal A., Oleszek W. (2014). Three new triterpene saponins from roots of *Eryngium planum*. Nat. Prod. Res..

[8] Kholkhal W., Ilias F., Bekhechi C., Bekkara F.A. (2012). *Eryngium maritimum*: A rich medicinal plant of polyphenols and flavonoids compounds with antioxidant, antibacterial and antifungal activities. Curr. Res. J. Biol. Sci..

[9] Mejri H., Tir M., Feriani A., Ghazouani L., Allaqui M.S., Saidani-Tounsi M. (2017). Does *Eryngium maritimum* seeds extract protect against CCl_4_ and cisplatin induced toxicity in rats: Preliminary phytochemical screening and assessment of its in vitro and in vivo antioxidant activity and antifibrotic effect. J. Funct. Foods.

[10] Hiller K., Pohl B., Franke P. (1981). Flavonoid spectrum of *Eryngium maritimum* L. Part 35. Components of some Saniculoideae. Pharmazie.

[11] Dinan L., Savchenko T., Whiting P. (2001). On the distribution of phytoecdysteroids in plants. CMLS, Cell. Mol. Life Sci..

[12] Adrian-Romero M., Wilson S.J., Blunden G., Yang M.-H., Carabot-Cuervo A., Bashir A.K. (1998). Betaines in coastal plants. Biochem. Syst. Ecol..

[13] Machado M., Santoto G., Sousa M.C., Salgueiro L., Cavaleiro C. (2010). Activity of essential oils on the growth of *Leishmania infantum* promastigotes. Flavour Fragr. J..

[14] Machado M., Sousa M.C., Salgueiro L., Cavaleiro C. (2010). Effects of essential oils on the growth of *Giardia lamblia* trophozoites. Nat. Prod. Commun..

[15] Darriet F., Znini M., Majidi L., Muselli B., Hammouti B., Bouyanzer A., Costa J. (2013). Evaluation of *Eryngium maritimum* essential oil as environmentally friendly corrosion inhibitor for mild steel in hydrochloric acids solution. Int. J. Electrochem. Sci..

[16] Darriet F., Bendahou M., Desjobert J.-M., Costa J., Muselli A. (2012). Bicyclo[4.4.0]decane oxygenated sesquiterpenes from *Eryngium maritimum* essential oil. Planta Medica.

[17] Darriet F., Andreani S., De Cian M.-C., Costa J., Muselli A. (2014). Chemical variability and antioxidant activity of *Eryngium maritimum* L. essential oils from Corsica and Sardinia. Flavour Fragr. J..

[18] Maggio A., Bruno M., Formisano C., Rigano D., Senatore F. (2013). Chemical composition of the essential oils of three species of Apiaceae growing wild in Sicily: *Bonannia graeca*, *Eryngium maritimum* and *Opopanax chironium*. Nat. Prod. Commun..

[19] Lajnef H.B., Ferioli F., Pasini F., Politowicz J., Khaldi A., D’Antuono F., Caboni M.F., Nasri N. (2017). Chemical composition and antioxidant activity of the volatile fraction extracted from air-dried fruits of Tunisian *Eryngium maritimum* L. ecotypes. J. Sci. Food Agric..

[20] Gygax A., Bernhardt K.G., Jogan N., Montagnani C., Gigot G. (2011). Eryngium Alpinum. https://www.iucnredlist.org/species/162328/5574460.

[21] Kikowska M., Kruszka D., Derda M., Thiem B. (2020). Phytochemical screening and acanthamoebic activity of shoots from in vitro cultures and in vivo plants of *Eryngium alpinum* L.—An endangered and protected species. Molecules.

[22] Le Clarie E., Schwaiger S., Banaigs B., Stuppner H., Gafner F. (2005). Distribution of a new rosmarinic acid derivative in *Eryngium alpinum* L. and another Apiaceae. J. Agric. Food Chem..

[23] Kikowska M., Thiem B., Szopa A., Klimek-Szczykułowicz M., Rewers M., Sliwinska E., Ekiert H. (2019). Comparative analysis of phenolic acids and flavonoids in shoot cultures of *Eryngium alpinum* L.—An endangered and protected species with medicinal value. Plant Cell Tiss. Organ Cult..

[24] Crowden R.K., Harborne J.B., Heywood V.H. (1969). (1969) Chemosystematics of the Umbelliferae—A general survey. Photochemistry.

[25] Dunkić V., Vuko E., Bezic N., Kremer D., Ruscic M. (2013). Composition and antiviral activity of the essential oils of *Eryngium alpinum* and *E. amethystinum*. Chem. Biodivers..

[26] Hegnauer R. (1973). Chemotaxonomie der Pflanzen.

[27] Hegnauer R. (1990). Chemotaxonomie der Pflanzen.

[28] Verpoorte R., Contin A., Memelnik J. (2002). Biotechnology for the production of plant secondary metabolites. Phytochem. Rev..

[29] Tasheva K., Kosturkova G., Petre M. (2013). Role of biotechnology for protection of endangered medicinal plants. Environmental Biotechnology—New Approaches and Prospective Applications.

[30] Thiem B., Kikowska M., Kurowska A., Kalemba D. (2011). Essential oil composition of the different parts and in vitro shoot cultures of *Eryngium planum* L.. Molecules.

[31] Pala-Paul J., Usano-Alemany J., Soria A.C., Perez-Alonso M.J., Brophy J.J. (2008). Essential oil composition of *Eryngium campestre* L. growing in different soil types. A preliminary study. Nat. Prod. Commun..

[32] Celik A., Aydinlik N., Arslan I. (2011). Phytochemical constituents and inhibitory activity towards methicillin-resistant *Staphylococcus aureus* strains of *Eryngium* species (Apiaceae). Chem. Biodivers..

[33] Cianfaglione K., Cianfaglione K., Blomme E.E., Quassinti L., Bramucci M., Lupidi G., Dall′Acqua S., Maggi F. (2017). Cytotoxic essential oils from *Eryngium campestre* and *Eryngium amethystinum* (Apiaceae) growing in central Italy. Chem. Biodivers..

[34] Matejić J.S., Stojanović-Radić Z.Z., Ristić M.S., Veselinović J.B., Zlatković B.K., Marin P.D., Džamić A.M. (2018). Chemical characterization, in vitro biological activity of essential oils and extracts of three *Eryngium* L. species and molecular docking of selected major compounds. J. Food Sci. Technol..

[35] Abou-Jawdah Y., Sobh H., Salameh A. (2012). Antimycotic activities of selected plant flora, growing wild in Lebanon, against phytopathogenic fungi. J. Agric. Food Chem..

[36] Thiem B., Goślińska O., Kikowska M., Budzianowski J. (2010). Antimicrobial activity of three *Eryngium* L. species (Apiaceae). Herba Pol..

[37] Kikowska M., Długaszewska J., Kubicka M.M., Kędziora I., Budzianowski J., Thiem B. (2016). In vitro antimicrobial activity of extracts and their fractions from three *Eryngium* L. species. Herba Pol..

[38] Meot-Duros L., Le Floch G., Magne C. (2008). Radical scavenging, antioxidant and antimicrobial activities of halophytic species. J. Ethnopharmacol..

[39] Murashige T., Skoog F. (1962). A revised medium for rapid growth and bioassays with tobacco tissue cultures. Physiol. Plant..

[40] Kikowska M., Thiem B., Szopa A., Ekiert H. (2020). Accumulation of valuable secondary metabolites: Phenolic acids and flavonoids in different in vitro systems of shoot cultures of the endangered plant species—*Eryngium alpinum* L. Plant Cell Tiss. Organ Cult..

[41] Adams R.P. (2007). Identification of Essential Oil Components by Gas Chromatography/Mass Spectroscopy.

[42] Joulain D., König W.A. (1998). The Atlas of Spectral Data of Sesquiterpene Hydrocarbons. Journal of Natural Products.

[43] Rodriques-Tudela J.L., Donnelly J.P., Arendrup M.C., Arikan-Akdagli S., Barchiesi F., Bille J., Chryssanthou E., Cuenca-Estrella M., Dannaoui E., Denning D. (2008). EUCAST Technical Note on the method for the determination of broth dilution minimum inhibitory concentrations of antifungal agents for conidia–forming moulds. Clin. Microb. Infection.

